# A 10-year analysis of thyrotoxic periodic paralysis in 135 patients: focus on symptomatology and precipitants

**DOI:** 10.1530/EJE-13-0381

**Published:** 2013-11

**Authors:** Chin-Chun Chang, Chih-Jen Cheng, Chih-Chien Sung, Tzong-Shi Chiueh, Chien-Hsing Lee, Tom Chau, Shih-Hua Lin

**Affiliations:** 1 Division of Nephrology, Department of MedicineTri-Service General HospitalNational Defense Medical Center No 325, Section 2, Cheng-Kung Road, Neihu 114, TaipeiTaiwan; 2 Division of Clinical Pathology, Department of PathologyTri-Service General HospitalNational Defense Medical Center No 325, Section 2, Cheng-Kung Road, Neihu 114, TaipeiTaiwan; 3 Division of Endocrinology and Metabolism, Department of MedicineTri-Service General HospitalNational Defense Medical Center No 325, Section 2, Cheng-Kung Road, Neihu 114, TaipeiTaiwan; 4 Department of MedicineProvidence St Vincent Medical CenterPortland, OregonUSA

## Abstract

**Background:**

A comprehensive analysis has not been performed on patients with thyrotoxic periodic paralysis (TPP) characterized by acute hypokalemia and paralysis in the setting of thyrotoxicosis.

**Purpose:**

The aim of this study was to analyze the detailed symptomatology of thyrotoxicosis and precipitating factors for the attack in a large cohort of TPP patients.

**Patients and methods:**

A prospective observational study enrolled patients with TPP consecutively over 10 years at an academic medical center. Clinical features, including signs/symptoms of thyrotoxicosis and precipitating factors, were analyzed. The Wayne's index was used to assess the severity of thyrotoxicosis at presentation. Patients who agreed to receive an oral glucose-loading test after recovery were evaluated.

**Results:**

Among the 135 TPP patients (male:female, 130:5), 70% of paralytic attacks occurred in the morning, especially during the seasons of summer and fall. Two-thirds of patients did not have a known family or personal history of hyperthyroidism. Only 17% of TPP patients manifested overt signs/symptoms of thyrotoxicosis (Wayne's index >19). A clear precipitating factor, such as high carbohydrate load, acute upper respiratory tract infection, strenuous exercise, high-salt diet, or the use of steroids or bronchodilators, was identified in only 34% of TPP patients. A glucose load to stimulate insulin secretion induced acute hypokalemia (K^+^2.47±0.6 mmol/l) with reparalysis in only 18% (10/55) of TPP patients.

**Conclusions:**

Most TPP patients have only subtle clinical signs/symptoms of thyrotoxicosis and only a small fraction has clear precipitating factors. In addition to the effects of hyperinsulinemia, other insulin-independent mechanisms may participate in the pathogenesis of TPP.

## Introduction

The spectrum of muscle weakness to paralysis induced by hypokalemia is called hypokalemic paralysis (HP), and the leading cause of hypokalemia-related medical emergencies (1). The etiology of HP can be generally classified into two groups: hypokalemic periodic paralysis (hypoPP), due to shift of potassium (K^+^) into the intracellular space without a total K^+^ deficit, and non-hypoPP, due to a large K^+^ deficit via gastrointestinal or renal loss (2). Among the hypoPP, familial hypokalemic periodic paralysis (FPP) is the most common cause in Western countries, and thyrotoxic periodic paralysis (TPP), characterized by the triad of acute hypokalemia without total body K^+^ deficit, muscle paralysis, and thyrotoxicosis, is the most common cause in Asia. However, with globalization and immigration, TPP is no longer confined to certain geographic areas (3, 4, 5, 6).

TPP is fraught with diagnostic and therapeutic challenges (2, 3, 7, 8, 9). It is crucial to recognize and treat early to avoid the life-threatening complications such as cardiac arrhythmias and respiratory failure. We have previously reported several diagnostic clues of TPP, including adult males without a family history of periodic paralysis exhibiting systolic hypertension, tachycardia, high QRS voltage or first-degree atrioventricular block on electrocardiography, as well as typical acid–base and electrolyte findings such as normal blood acid–base status, hypokalemia with low urinary K^+^ excretion, hypophosphatemia with hypophosphaturia, and hypercalciuria (1, 2, 3, 10, 11, 12). With respect to emergent therapy, the dose of KCl should be minimized to avoid rebound hyperkalemia. For patients who are ‘refractory’ to KCl treatment, non-selective β-blockers may be an alternative. These patients often manifest persistently decreased serum K^+^ concentration after KCl supplement associated with evidences of hyperadrenergic activity (13).

There are several unresolved issues in TPP. It is often stated in the literature that typical clinical symptoms of thyrotoxicosis, such as weight loss, heat intolerance, palpitations, increased appetite, excitability, and diaphoresis, may be subtle and periodic paralysis may precede these symptoms in TPP (10, 14, 15, 16). However, the exact severity of thyrotoxicosis has not been evaluated in TPP patients at the time of presentation. In terms of precipitating factors, several endogenous and exogenous factors that stimulate the Na^+^–K^+^-ATPase have been reported to precipitate attacks of TPP (17, 18, 19). High carbohydrate loads and strenuous exercise are well-recognized precipitating factors for TPP as well as FPP. Nevertheless, the exact mechanism and relative contribution of each of these factors to the pathogenesis of TPP is still unclear. We have collected a large set of phenotypic and biochemical data from TPP patients over the past 10 years. In this study, we analyzed these patients focusing on symptomatology and precipitating factors, in order to provide a better understanding of its clinical features and pathogenesis. Glucose-loading test was also performed in a considerable number of these patients to evaluate the role of hyperinsulinemia in triggering attacks.

## Subjects and methods

### Subjects

**Figure 1:**
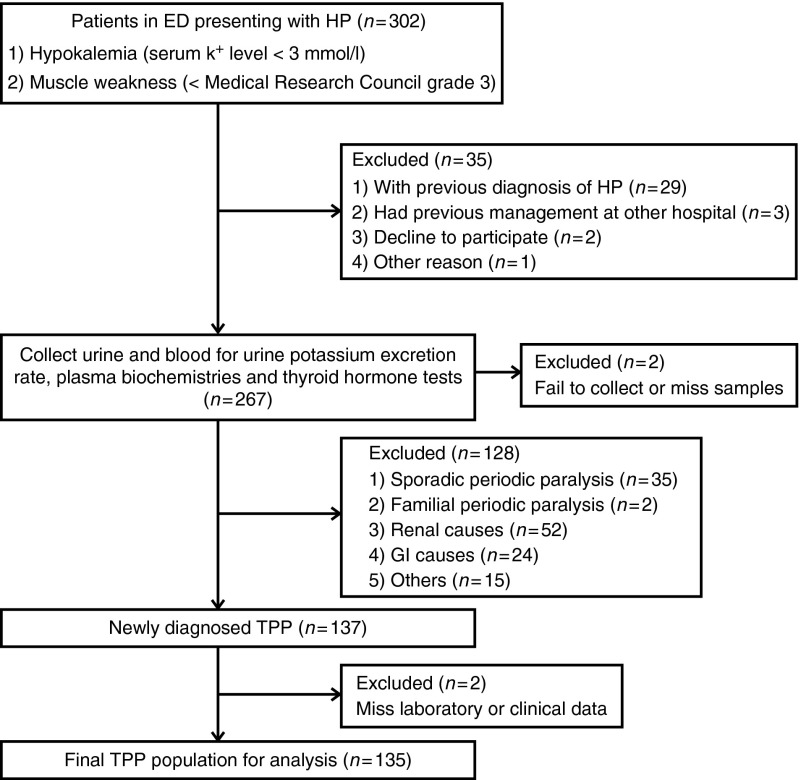
Flow diagram of assessment for eligibility. ED, emergency department; HP, hypokalemic paralysis; GI, gastrointestinal; TPP, thyrotoxic periodic paralysis.

The study protocol was approved by the Ethics Committee on Human Studies at Tri-Service General Hospital, National Defense Medical Center, in Taiwan. From January 2002 to December 2012, the patients presenting with hypokalemia (serum K^+^ concentration <3.0 mmol/l) and acute muscle weakness in the emergency department (ED) were visited, examined, and collected by at least an experienced nephrologist. The process of the participants' selection is shown in a flow diagram (Fig. 1). The diagnosis of TPP was confirmed by fulfilling the following criteria: low urine K^+^ excretion rate (transtubular potassium gradient <3) and thyrotoxicosis confirmed by thyroid function tests – low thyroid-stimulating hormone (TSH) and high tri-iodothyronine (T_3_) and free thyroxine (FT_4_). Other causes of HP, such as renal causes and abuse of diuretics and laxatives, were excluded. Patients with previously diagnosed TPP, concomitant medication that confounds the interpretation of renal K^+^ excretion and thyroid function tests, missing blood or urine sample collections, or referred from other hospitals after medical treatment were excluded. The history inquiry, physical examination, laboratory tests, and KCl supplementation were performed as previously reported (2, 10, 11, 12, 13). The clinical features, signs, and symptoms of thyrotoxicosis and precipitating factors were recorded.

### Clinical features and laboratory measurement

Historical and demographic data were collected on each TPP patient, including gender, age, height, body weight, vital signs, family history of periodic muscle paralysis and hyperthyroidism, personal history of hyperthyroidism, and time-of-onset of the paralytic attack. Hemodynamic monitoring and laboratory examinations were performed as previously reported (2, 10, 11, 12, 13). Blood gases were measured with an ABL 510 (Radiometer, Copenhagen, Denmark). Blood biochemical values and electrolytes were determined with the use of automated methods (AU 5000 chemistry analyzer; Olympus, Tokyo, Japan). Thyroid function tests were measured by RIAs (Diagnostic Products Corporation, Los Angeles, CA, USA). The estimated glomerular filtration rate (eGFR) was calculated using the Modification of Diet in Renal Disease (MDRD) formula. To define the underlying causes of hyperthyroidism, TPP patients received the measurement of serum thyroglobulin, thyrotropin receptor antibody, thyroid ultrasound, and iodine-131 thyroid scan.

### Signs and symptoms of thyrotoxicosis

The Wayne's index was used to quantitatively evaluate the severity of signs and symptoms of thyrotoxicosis at initial presentation of all TPP patients. This index, albeit old, has demonstrated accuracy in the clinical assessment and diagnosis of thyrotoxicosis (20, 21). It scores the patient on nine symptoms (palpitations, preference for heat, preference for cold, appetite change, weight change, diaphoresis, fatigue, dyspnea on exertion, and nervousness) and ten signs (palpable thyroid, bruit over the thyroid gland, exophthalmos, lid retraction, lid lag, hyperkinetic movements, fine finger tremor, hot and moist hands, tachycardia, and atrial fibrillation). Of a total score of 45, >19 is considered thyrotoxic, 11–19 equivocal, and <11 euthyroid.

### Precipitating factors

The precipitating factors of TPP reported in the literature included high carbohydrate ingestion, strenuous exercise, trauma, acute upper respiratory tract infection (URI), high-salt diet, emotional stress, exposure to cold, alcohol ingestion, menstruation, and use of drugs such as corticosteroids, epinephrine, acetazolamide, and non-steroidal anti-inflammatory drugs (2, 3, 14, 22, 23, 24). These factors precede the acute attack of TPP <24 h and strongly associate with the disease mechanism of TPP. Our patients were questioned on all these factors, and their carbohydrate and salt ingestion was based on single 24-h dietary recall (25, 26). We also evaluated the available medical records for each patient to identify the use of the above-mentioned drugs that might have been overlooked by patients.

### Glucose-induced paralysis

To evaluate whether high carbohydrate loads predisposed patients to acute hypokalemia and muscle paralysis via hyperinsulinemia, patients were asked to receive an oral glucose challenge (2 g/kg), modified from a protocol previously described (27), 1 day after full recovery. TPP patients who agreed underwent blood sampling prior to and every hour after the glucose load over a 3-h period. Their muscle strength and ECG were closely monitored and serum electrolytes and insulin were measured hourly. Oral or i.v. KCl supplementation (10 mmol/h) was given to those who developed acute hypokalemia and paralysis until muscle recovery.

### Statistical analysis

Results are expressed as mean±s.d. A one-way ANOVA was used to compare plasma FT_4_ levels among the three grades of thyrotoxicosis by the Wayne's index. The relationship between initial serum K^+^ and the FT_4_ concentration was analyzed by simple linear regression. The unpaired Student's *t*-test was used to determine significant differences in variables between TPP patients with and without induced HP in glucose-loading tests at the *P* value <0.05 level of confidence. The Mann–Whitney *U* test was used when the variables between two subgroups were not normally distributed. Analyses were performed using PASW Statistics18 (SPSS 18.0) statistical software.

## Results

### Patients' characteristics

Over 10 years, there were 302 patients presenting with acute hypokalemia and muscle paralysis in our ED. Totally, 135 TPP patients were enrolled in this study according to our protocol (Fig. 1). Their characteristics are shown in Table 1. They had a remarkable male predominance (male:female, 26:1) and young age of onset (mean 30.6 years, range 16–62 years) with 80% in the 20- to 40-year age group. Their thyroid function showed high T_3_ (244.0±95.1 ng/dl), high FT_4_ (4.5±2.8 ng/dl), and suppressed TSH level (all <0.03 mU/l). Graves' disease with a positive thyrotropin receptor antibody, a diffuse enlargement of the thyroid gland, and/or diffusely increased radioiodine uptake in the thyroid accounted for the major cause of hyperthyroidism (130/135, 96.3%). Other causes of thyrotoxicosis included the use of T_3_-containing weight-reducing agents in two, subacute thyroiditis in one, toxic multi-nodular goiter in one, and toxic single nodular goiter in one. Twenty-nine patients (21.5%) had a family history of hyperthyroidism in at least one first-degree family member and 33 patients (24.4%) had already been diagnosed with hyperthyroidism prior to their first attack of paralysis. Seventeen (12.6%) TPP patients had both family and personal histories of hyperthyroidism. No patient had a family history of periodic paralysis. With respect to the timing of paralytic attacks, most of them occurred in the morning (0400–1200 h; 95/135, 70%) followed by evening (1700–2300 h; 26/135, 19%). Most TPP attacks occurred in the summer (35%, June–August) and fall (30%, September–November). In addition to the typical clinical and laboratory features mentioned earlier (in the Introduction), hypocreatininemia (0.6±0.15 mg/dl) with high eGFR (168.1±37.8 ml/min per 1.73 m^2^) due to the increased renal blood flow related to hyperthyroidism was also very common in TPP patients.

All our TPP patients received non-selective β-blockers for the prevention of the attack and oral KCl supplement (8–16 mmol/h) only if they had recurrent acute muscle symptoms. The definite control of hyperthyroidism is the key to prevent the recurrence of TPP. Anti-thyroid agents were given shortly after the diagnosis of hyperthyroidism for most of our TPP patients with Graves' disease. Approximately 5% (6/130) of them received surgical thyroidectomy (2/130) or oral radioactive iodine-131 ablation therapy (4/130) because of uncontrollable Graves' disease. For the other abovementioned causes of thyrotoxicosis, there was no recurrence of TPP attack after the discontinuation of offending drugs, spontaneous resolution of thyroiditis, and the medical treatment with anti-thyroid agents followed by surgical thyroidectomy for toxic nodular goiter. Overall, ∼30% (40/135) of our TPP patients had recurrent attacks, mostly during the withdrawal or tapering of the anti-thyroid agents.

### Signs and symptoms of thyrotoxicosis

**Figure 2:**
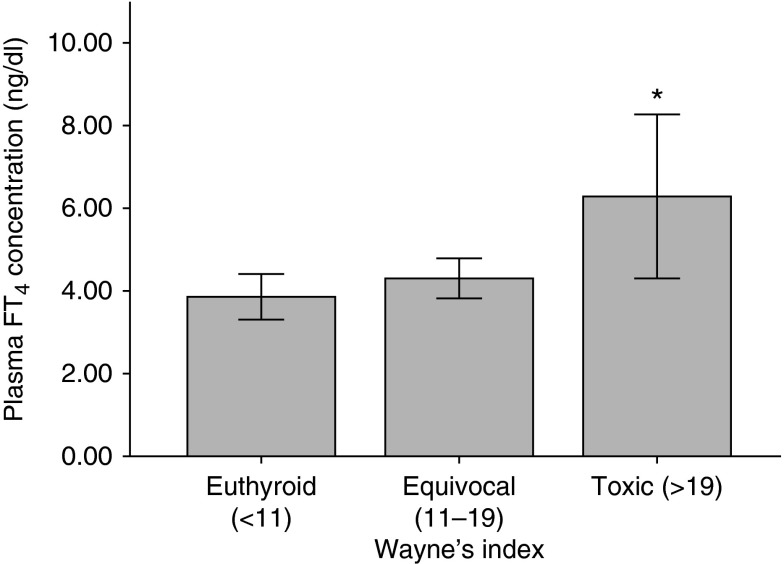
Plasma FT_4_ concentration in TPP patients with different Wayne's indices. *P<0.01, toxic vs euthyroid.

Most TPP patients were clinically equivocal (45%, Wayne's index score 11–19) or even euthyroid (38%, Wayne's index score <11). Only 17% manifested overt signs and symptoms of hyperthyroidism (Wayne's score >19). Furthermore, only 16% of the two-thirds of TPP patients who had neither personal nor family history exhibited overt thyrotoxicosis. TPP patients with toxic signs/symptoms of hyperthyroidism had higher plasma levels of FT_4_ than the clinically euthyroid patients (Fig. 2). However, there was no significant correlation between the initial serum K^+^ and FT_4_ concentration (*P*>0.05).

### Precipitating factors

We found identifiable precipitating factors of attacks in 34% (46/135) of TPP patients. Of these, most (41/46, 89%) had a single precipitant; five patients (5/46, 11%) had two coexisting precipitating events. The identified precipitating events for TPP attacks were high carbohydrate ingestion (16/135, 12%), recent acute URI (11/135, 8%), strenuous exercise (10/135, 7%), high-salt diet (4/135, 3%), trauma (1/135, 1%), and use of corticosteroids (1/135, 1%) and β-adrenergic bronchodilators (3/135, 2%). All the TPP patients with more than one precipitant had carbohydrate-rich food ingestion, four overlapped with exercise and one with acute URI. There were no significant differences in clinical course between TPP patients with single and multiple precipitants. The clinical characteristics and biochemical studies were also not statistically different between TPP patients with and without identifiable precipitating events.

### Glucose-induced paralysis

**Figure 3:**
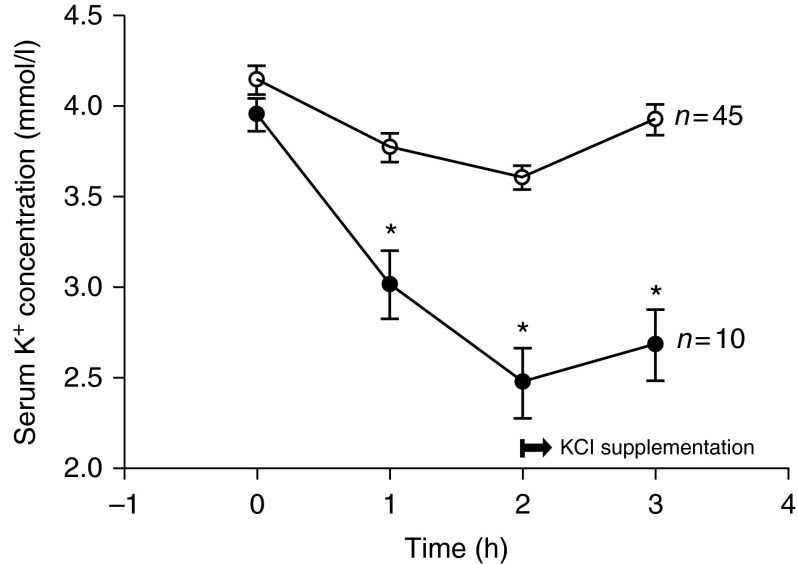
Serum K^+^ concentration in 55 TPP patients with (closed circle) and without (open circle) glucose-induced paralysis. *P<0.01.

Fifty-five TPP patients agreed to an oral glucose-loading test after recovery. Only ten patients (10/55, 18%) redeveloped acute hypokalemia and muscle paralysis. They often had an abrupt fall of their serum K^+^ concentration at 1 h and a nadir in the second hour with muscle weakness (muscle power <3) (Fig. 3). All of them recovered muscle power within 6–8 h after prompt oral or i.v. KCl supplementation. None of them developed serious complications, such as acute respiratory failure or ventricular arrhythmia. Three of ten (30%) TPP patients who demonstrated provocative paralysis had an identifiable precipitating factor (two with prior ingestion of sweets and one with recent URI). Eleven of the remaining 45 (24%) patients without provocable attacks had identifiable predisposing events (four with recent URI, three with ingestion of carbohydrate-rich food, two with strenuous exercise, and two with concurrent exercise and ingestion of carbohydrate-rich food). There was no significant difference in gender, age, BMI, blood pressure, heart rate, precipitants, and biochemical studies, including plasma insulin, between TPP patients with and without provocable attacks (Table 2).

## Discussion

This prospective study provides clinical insight, focusing on symptoms related to hyperthyroidism and precipitants, in a large cohort of TPP in Taiwan. Most attacks of TPP occurred in the morning and during the seasons of summer and fall. Two-thirds of TPP patients did not have known personal or family histories of hyperthyroidism. Using Wayne's index, we demonstrated that only 17% (23/135) of TPP patients had signs and symptoms of thyrotoxicosis. Furthermore, only one-third of TPP patients had identifiable precipitating factors. Similarly, glucose challenge stimulating endogenous hyperinsulinemia provoked HP in only 18% (10/55) of TPP patients.

TPP patients in this study had remarkable male predominance (male-to-female ratio of 26:1) despite the fact that hyperthyroidism is more common in females (female-to-male ratio of 9:1). This finding also existed in our previous report and other observational studies. Androgens, muscle mass, and serum catecholamine levels in response to stress may explain the gender difference (28, 29, 30, 31, 32). Androgens have been reported to increase the expression and activity of the Na^+^, K^+^-ATPase (28, 29, 33). Testosterone enhances hypertrophy of myoblasts and thus causes higher muscle-to-body mass ratio and total Na^+^, K^+^-ATPase abundance in males (30, 34). Catecholamines, a strong activator of Na^+^, K^+^-ATPase activity, have been found to be released more in males in response to experimental stress (31, 32). The preponderance of TPP attacks in the morning may also stem from higher plasma catecholamines and sympathetic tone in the late morning (35). With respect to the seasonal variation, increased outdoor activity with increased perspiration of K^+^ and ingestion of sugar-containing cold beverages may be responsible for the higher prevalence of TPP attacks in the summer and fall in Taiwan.

Thyrotoxicosis is a prerequisite for the diagnosis of TPP. Because thyroid hormone measurement is not often available in the ED, prompt diagnosis of thyrotoxicosis primarily relies on the family and personal histories and clinical assessment of hyperthyroid signs/symptoms. In this study, only one-third of TPP patients had known personal or family history of hyperthyroidism. According to previous reports, nearly 50% of TPP patients had only subtle signs/symptoms of thyrotoxicosis, albeit without systematic assessment (14, 15, 16, 36). Using the Wayne's index, a reliable quantitative score of hyperthyroid severity (20, 21), we found that only 17% of TPP patients had toxic thyrotoxicosis (score >19), supporting the notion that most TPP patients have equivocal symptoms. This finding was consistent across patients with and without personal/family histories of hyperthyroidism. For those (76/135, 56.3%) who did not have known personal/family history or overt clinical thyrotoxicosis, several clinical and laboratory findings characteristic of TPP, mentioned in the Introduction, are helpful in making the rapid diagnosis of TPP (1, 2, 3, 10, 11, 12).

Precipitating factors, including high carbohydrate loads, strenuous exercise, trauma, acute URI, high-salt diet, emotional stress, exposure to cold, alcohol ingestion, menstruation, and use of drugs such as corticosteroids, epinephrine, acetazolamide, and non-steroidal anti-inflammatory drugs, have been reported with attacks of TPP. In reviewing these factors with our TPP patients in detail, we found that only the minority of them (34%) had identifiable precipitants. TPP patients with and without known precipitating factors had no significant differences in their clinical and biochemical findings. Our study also showed that high carbohydrate loads and strenuous exercise, the two most-recognized precipitating factors in TPP and FPP (14, 34), were implicated in only 12 and 7% of our patients respectively. The low frequency of high-carbohydrate-provoked TPP in our observation may be related to the westernization of the Asian diet with declining rice intake and increasing protein consumption (14, 22). Nevertheless, other potential provocative factors still need to be scrutinized in TPP patients without currently identifiable precipitants.

The role of insulin in TPP was revealed by the finding that hyperinsulinemia was observed during acute attacks of TPP and that TPP patients had exaggerated insulin responses to oral glucose challenge in comparison with patients with pure hyperthyroidism (17, 18, 19). The hyperinsulinemic response may account for the association of TPP with high-carbohydrate ingestion. To further examine the role of endogenous hyperinsulinemia in precipitating paralytic attacks, we performed an oral glucose-loading test (2 g/kg) during the day, without prior fast, mimicking the typical ingestion of high-carbohydrate meals, sweet snacks, or sugar-containing drinks. In contrast to previous reports (15, 37, 38), only a small portion (18%, 10/55) of our TPP patients developed acute hypokalemia with paralysis after glucose challenge. TPP patients with provocable attacks had similar insulin responses to those without induced paralysis. This finding is relatively similar to a previous study showing that 29% (2/7) of TPP patients had glucose-induced paralysis (36) and a more recent study reporting no paralytic attacks in 51 TPP patients during oral glucose tolerance tests (19). Based on the infrequency of glucose load-induced paralysis, other insulin-independent factors must also be involved in the pathogenesis of TPP.

Acute hypokalemia, the principle laboratory finding in TPP, correlates with the severity of paralysis and resolves when normal muscle strength returns. Extracellular K^+^ homeostasis is primarily controlled by the Na^+^, K^+^-ATPase and K^+^ channels in the skeletal muscles, which provide the main access for inward and outward K^+^ movements respectively. Increased Na^+^, K^+^-ATPase activity has traditionally been implicated in the pathogenesis of TPP. Because only a minority (∼2%) of patients with hyperthyroidism develops TPP, activation of the Na^+^, K^+^-ATPase through thyroid hormone-mediated mechanisms cannot be the sole cause. In addition, increased Na^+^, K^+^-ATPase activity in skeletal muscle can be compensated by increased K^+^ efflux. In fact, insulin and catecholamines not only activate Na^+^, K^+^-ATPase but may also inhibit inward rectifying K^+^ (Kir) channels (39). Recent studies have shown that susceptibility to TPP can be conferred by loss-of-function mutations in the skeletal muscle-specific Kir channel, *Kir2.6* (40, 41, 42), and loci on 17q involved in Kir2.1 gene expression (43, 44, 45). These findings also suggest that reduced basal muscular Kir current may also be an important mechanism of TPP (46). The dual hits of increased intracellular K^+^ influx from activated Na^+^, K^+^-ATPase and decreased K^+^ efflux from defective Kir channels potentiates the serum hypokalemia that upsets membrane polarization and muscle excitability in TPP (46). The role of reduced K^+^ channels should be further verified in our TPP patients.

There are some limitations in this study. First, we did not include the thyrotoxic patients without paralysis as disease control. The comparison between thyrotoxic patients with and without paralysis yields the potential risk factors and disease mechanisms of TPP. Nevertheless, the aim of this study focused on the symptomatology of thyrotoxicosis and the precipitating factors of TPP. Secondly, many of the predisposing factors could not be quantified. For example, we did not quantify the amount of carbohydrate intake to avoid reporting error and define the high carbohydrate intake as the precipitating factor. However, we have performed oral glucose tests to simulate the effect of high-carbohydrate diet on the attack of paralysis.

In conclusion, TPP patients often have subtle signs and symptoms of thyrotoxicosis on presentation of paralysis. In contrast to standard teaching, most TPP patients also do not have clear precipitating factors or glucose load-induced paralytic attacks. Besides hyperinsulinemia, other insulin-independent factors may also play pivotal roles in the pathogenesis of TPP.

## Figures and Tables

**Table 1:** Clinical and laboratory features of 135 TPP patients.

**Study**	**Reference range**	**Result**
Clinical feature		
Gender (male/female)	NA	130:5
Age (years)	NA	30.6±8.2
BMI (kg/m^2^)	18.5–24.9	25.3±3.6
Systolic blood pressure (mmHg)	NA	140.0±14.4
Diastolic blood pressure (mmHg)	NA	76.1±10.4
Heart rate (beats/min)	NA	98.8±14.5
Plasma		
TSH (μU/ml)	0.25–5.00	<0.03
Tri-iodothyronine (ng/dl)	86.0–187.0	244.0±95.1
Free thyroxine (ng/dl)	0.8–2.0	4.5±2.75
Na^+^ (mmol/l)	136–145	139±3
Initial K^+^ (mmol/l)	3.5–5.1	2.17±0.4
Cl^−^ (mmol/l)	98–107	106±2
pH	7.35–7.45	7.40±0.02
Bicarbonate (mmol/l)	22.0–26.0	23±2
Ionized calcium (mg/dl)	4.5–5.3	4.81±0.22
Phosphate (mg/dl)	2.7–4.5	2.34±0.77
Creatinine (mg/dl)	0.7–1.2	0.60±0.15
eGFR (ml/min per 1.73 m^2^)	90–120	168.1±37.8

**Table 2:** Comparison between TPP patients with and without glucose-induced paralysis.

**Study**	**Reference range**	**Paralysis** (*n*=10)	**Non-paralysis** (*n*=45)
Clinical feature			
Gender (male/female)	NA	10:0	44:1
Age (years)	NA	31.1±7.8	31.8±9.6
BMI (kg/m^2^)	NA	25.9±2.3	24.9±3.4
Systolic blood pressure (mmHg)	NA	131.2±11.5	133.8±15.6
Diastolic blood pressure (mmHg)	NA	73.2±7.3	76.7±9.9
Heart rate (beats/min)	NA	100.3±19.8	104.26±12.6
Precipitating factors (+)	NA	3/10	11/45
Plasma			
K^+^ on paralysis (mmol/l)	3.5–5.1	2.47±0.6*	3.60±0.3
TSH (μU/ml)	0.25–5.00	<0.03	<0.03
Tri-iodothyronine (ng/dl)	86.00–187.00	272.2±68.9	210.9±76
Free thyroxine (ng/dl)	0.80–2.00	3.74±0.9	3.8±1.3
Creatinine (mg/dl)	0.7–1.2	0.6±0.1	0.6±0.1
eGFR (ml/min)	90–120	166.4±37.6	168.3±46.6
Peak insulin (μIU/ml)	NA	135.6±60.6	122.1±48.1

*P value <0.01.
